# Stress and loneliness in marriage: does loneliness moderate the effects of daily stress on marital satisfaction?

**DOI:** 10.3389/fpsyg.2026.1871430

**Published:** 2026-07-09

**Authors:** Nick Frye-Cox, Jeremy B. Yorgason

**Affiliations:** 1 Human Sciences & Design, Baylor University, Waco, TX, United States; 2 School of Family Life, Brigham Young University, Provo, UT, United States

**Keywords:** couple (spouses), dyadic daily diary, loneliness, marital satisfaction, stress

## Abstract

Although stress is widely recognized as a key predictor of marital satisfaction, less is known about how daily experiences of loneliness may shape this association. Loneliness, whether stable or fluctuating, may alter how spouses interpret and respond to daily stressors. Participants were 155 heterosexual married couples who completed daily surveys over seven consecutive days. Using a dyadic intensive longitudinal design, participants reported daily levels of stress, loneliness, and marital satisfaction. Results from dyadic multilevel models revealed that, for wives, average loneliness moderated the between-person association of stress and marital satisfaction, among wives who reported lower loneliness, higher stress was associated with lower marital satisfaction, whereas this association did not reach statistical significance among wives with higher levels of loneliness. For husbands, within-person fluctuations of daily loneliness moderated the within-person association of stress on marital satisfaction, with loneliness amplifying the association between daily stress and marital satisfaction. Both stable and fluctuating experiences of loneliness may alter how stress is associated with marital satisfaction, but these associations may differ for husbands and wives.

## 1 Introduction

Whether navigating minor daily hassles or major life transitions, spouses’ experience of stress has been associated with both positive and negative relationship functioning (Neff and Williams, 2025; Ogan and Monk, 2025). The association between stress and marital quality may vary, suggesting that certain conditions may strengthen or weaken these associations. According to the Vulnerability-Stress-Adaptation model, enduring vulnerabilities such as attachment insecurity, low self-esteem, and neuroticism interact with acute and chronic stressors in ways that are associated relationship outcomes (Karney and Bradbury, 1995; McNulty et al., 2021). Loneliness may represent one such vulnerability.

Contrary to conventional wisdom, romantic relationships do not inherently protect against feelings of loneliness. Prior research has shown that approximately 45% of spouses may experience at least moderate levels of loneliness (Wilson and Moulton, 2010). Existing research generally shows that loneliness in marriage is negatively associated with relationship quality (Mund et al., 2022). Despite recent proliferation of work in this area, loneliness has predominately been conceptualized as a trait-like construct, despite accumulating evidence supporting its transitory nature (Buecker et al., 2024). How spouses’ fluctuations in loneliness correspond with marital processes remains unclear.

Given the importance of stress and loneliness for relationship outcomes, it is necessary to identify the conditions under which stress is more strongly associated with relationship functioning. Recent evidence suggests that both stress and loneliness are associated with more negative marital perceptions (e.g., Mund and Johnson, 2021; Neff and Karney, 2017). Achieving a greater understanding of how stress and loneliness may coincide with marital satisfaction may support efforts to develop effective marital interventions. Research to date, however, has yet to investigate the association between these constructs. Therefore, the present study examined the extent to which stress and loneliness interact in the combined association with marital satisfaction.

### 1.1 Stress in marriage

Stress, conceptualized as a psychological state that arises when environmental demands tax or exceed an individual’s resources (Lazarus and Folkman, 1984), plays a significant role in marital relationships. The experience of stress and its consequences are influenced by the broader relational context (Hobfoll et al., 2018; Randall and Bodenmann, 2017). Marriage represents one such relational context, characterized by interdependent processes in which one partner’s experiences are linked with the other partner’s thoughts and feelings about their marriage.

Prior research has demonstrated that stress may be associated with poorer relationship outcomes (Neff and Karney, 2017). Building on this body of work, researchers have drawn on the VSA model to explain associations between stress and marital functioning (Karney and Bradbury, 1995; McNulty et al., 2021). From this perspective, stress is linked with more negative spousal perceptions of marriage, with the strength of such association likely contingent on existing vulnerabilities that make some spouses more sensitive to stressful experiences. Empirical tests of the VSA model have generally relied on cross-sectional studies (e.g., Falconier et al., 2014; see also Randall and Bodenmann, 2017) or longitudinal panel studies with prolonged time between measurements (e.g., McNulty et al., 2021; Ogan et al., 2021). Although these designs have provided important insights, their reliance on retrospective reports are too far removed from the experience of stress to appropriately assess the extent to which stress may impact relationship evaluations (Cooper et al., 2019; Gorin and Stone, 2001). Given these measurement concerns, researchers have increasingly leveraged intensive longitudinal designs (e.g., daily diaries) to properly link stress with relationship processes and outcomes.

Studies using daily diary data generally have shown that daily stressors may indeed be linked with poorer relationship outcomes, primarily though stress spillover and crossover effects (Neff and Karney, 2017; Totenhagen et al., 2023). Totenhagen et al. (2012) found that daily external stress is associated with increased distance and reduced closeness between partners. Additionally, Neff and Buck (2023) demonstrated that individuals experiencing higher levels of stress became more attuned to their partner’s negative behaviors while showing no increased awareness of positive behaviors, suggesting that stress may coincide with biased perceptions of a partner’s behavior. Although this research has established the importance of examining daily stress processes in marriage, less is known about how experiences of loneliness may coincide with stress and relationship outcomes.

### 1.2 Loneliness in marriage

Approximately one-third of adults older than 45 report chronic loneliness (Wilson and Moulton, 2010). Such elevated rates of loneliness among married couples may be attributed to spouses’ consolidation of their social networks and increased expectations for their spouse to fulfill their emotional and social needs (Finkel et al., 2014; Haggerty et al., 2023). When spouses’ high expectations for emotional connection remain unfulfilled, they may develop a sense of loneliness, that may contribute to lower levels of intimacy and marital quality (Frye-Cox and Hesse, 2013; Stevens and Westerhof, 2006). This reasoning aligns with both cognitive and evolutionary frameworks of loneliness that posit feelings of loneliness may arise from perceived discrepancies between desired and actual levels of social connection (Perlman and Peplau, 1981), which are theorized to motivate individuals to repair social relationships (Gardner et al., 2005; Hawkley and Cacioppo, 2010).

Research has generally shown that loneliness may be negatively associated with relationship outcomes. Longitudinal research by Mund and Johnson (2021) demonstrated that loneliness is associated with relationship satisfaction over time, with significant actor and partner effects persisting for over 8 years. Additional research that has explored the interpersonal dynamics that may underlie these associations has shown that loneliness appears to be more strongly associated with subjective relationship evaluations (e.g., perceived emotional closeness, relationship quality) than with more than quantifiable indicators of relationship behavior such as frequency of sex or physical expressions of affection (Mund et al., 2022). Furthermore, Lemay et al. (2024) found that lonely individuals tended to perceive lower levels of partner care and regard, with these perceptions corresponding to lower relationship satisfaction.

Although the direct associations between loneliness and relationship outcomes have been examined, it is also plausible that loneliness may moderate the link between stress and marital outcomes given that stress and loneliness tend to co-occur both concurrently and longitudinally (Laustsen et al., 2023). Understanding how these experiences operate within marriage has become increasingly important given their designation as public health concerns (American Psychological Association, 2023; U.S. Surgeon General, 2023). Thus, an investigation of the interactive association between stress and loneliness within marriage may provide a better understanding of this growing health concern.

In accord with the VSA model, we propose that loneliness may be positioned as a vulnerability, both at the between-person level and within-person level, that may qualify the association between stress and relationship outcomes. Recent longitudinal evidence suggests that stress and loneliness are likely to co-occur (Laustsen et al., 2023). Lonely individuals demonstrate heightened stress reactivity and prolonged emotional responses to daily stressors (Kang et al., 2024), suggesting that loneliness may mark a context in which stress is more strongly associated with marital quality. Given that both stress and loneliness are associated with lower cognitive resources (Buck and Neff, 2012; Wong et al., 2022), their concurrent experience may be particularly relevant for spouses’ engagement in relationship maintenance behaviors that promote marital functioning. The documented tendency of lonely individuals to exhibit negative biases in relationship evaluation (Lemay et al., 2024; Spithoven et al., 2017) could reflect that lonely spouses may perceive or use available partner support, particularly during times of stress.

Although much of the literature has examined loneliness as a trait analyzed at the between-person level, recent evidence also suggests that loneliness may vary from day-to-day (Buecker et al., 2024; Shao et al., 2026). At the between-person level, loneliness may operate as an enduring vulnerability, such that stress is expected to be more strongly associated with lower marital satisfaction among spouses who report higher average levels of loneliness. At the within-person level, daily loneliness may operate as a time-varying vulnerability, such that stress is expected to be more strongly associated with lower marital satisfaction on days when spouses report higher loneliness than usual. Indeed, research by Kang et al. (2024) has shown that lonely individuals demonstrate heightened stress reactivity and prolonged emotional responses to daily stressors (Kang et al., 2024), suggesting that higher loneliness may characterize spouses for whom daily stress is more strongly associated with lower marital satisfaction.

### 1.3 Sex differences in stress and loneliness

Empirical investigations of stress and loneliness are rife with attempts to establish systematic sex differences in relationship processes and outcomes. Research on sex differences in stress and loneliness is typically grounded in gender role theories which posit that factors such as cultural beliefs, norms, and societal expectations about gender are associated with differential experiences and outcomes (Pollitt and Curran, 2022; Thobejane and Khoza, 2014). In Western cultures, gender socialization processes may contribute to a communal orientation (e.g., social integration, other-focused) for women, whereas men may be socialized to adopt an agentic orientation (e.g., autonomy and self-related goals; Haines and Stroessner, 2019; Wojciszke et al., 2009), with such sex differences suspected to correspond to variations in stress and loneliness.

According to tend-and-befriend theory, stress is theorized to be associated with seeking connection among women, whereas men may rely on fight-or-flight responses that emphasize autonomy (Taylor et al., 2000). In support of this premise, past research has shown that women report higher levels of daily stress and more family-related stressful events compared to men, often citing relationship and family responsibilities as key stressors (Almeida and Kessler, 1998; American Psychological Association, 2023; Matud, 2004). To manage such stress, women’s ability to seek connection and support appears particularly dependent on perceiving their relationships as responsive and available. Loneliness may be linked with women’s perception that existing relationships can provide adequate support. Men’s fight-or-flight stress responses may be associated with autonomous coping strategies that minimize relational engagement (Nickels et al., 2017; Taylor et al., 2000), further limiting emotional availability and connection to a spouse (Frye-Cox and Hesse, 2013).

### 1.4 Current study

Drawing from stress and relationship theories, the present study examined the extent to which spouses’ daily experience of stress and loneliness interact in relation to their own as well as their partner’s marital satisfaction. We hypothesized that loneliness would moderate the negative effects of stress on marital satisfaction such that the association would be more pronounced among spouses who report higher average levels of loneliness (between-person) and on days characterized by higher levels of loneliness (within-person) for both spouses. We also explored potential gender differences in these associations.

Our study advances an understanding of stress and loneliness by capturing how these constructs may manifest as an enduring characteristic and as a state that fluctuates from day to day to coincide with spousal reports of marital satisfaction. Disentangling between-person differences from within-person daily fluctuations provides a better understanding of how stress and loneliness are associated with marital satisfaction for both husbands and wives (Hilpert et al., 2018). Such a nuanced approach can also help refine prominent theoretical frameworks that have been grounded in evidence analyzed at the between-person level. Because marital satisfaction is a key predictor of both relationship stability and individual health outcomes, identifying how daily experiences of stress and loneliness are linked with both partners’ satisfaction levels could inform more effective interventions for couples.

## 2 Materials and methods

### 2.1 Procedure and sample

Participants were recruited by students enrolled in a communication course at a large Midwestern university using a convenience sampling approach. Students were instructed to recruit participants who were at least 18 years old, had reliable access to an electronic device, were married, and were married to a spouse who was also willing to participate in the study. Students were instructed that couples were not eligible if at least one partner was unable to read English and complete the daily surveys. Students provided contact information for each recruited couple to the research team. A team member then contacted each couple to verify eligibility and explain the study purpose. After confirming eligibility, a team member emailed each spouse separately with instructions for completing the daily surveys.

Students received nominal extra credit for locating eligible participants. The study was reviewed and approved by an institutional review board. All participants were informed of the study procedures before providing informed consent.

Our study relied on interval contingent assessments in which married couples were asked to complete daily surveys at the end of each day for 8 consecutive days. More specifically, Day 1 served as a baseline measure in which data for demographics were collected, whereas the remaining days served as the primary sources of data for the dyadic daily diary analyses. Participants were emailed a unique link at 5:00 p.m. that provided access to the daily survey via SurveyMonkey. Participants were instructed to complete the surveys separately from their spouse before going to bed and that the daily link would expire at 11:59 p.m. At 8:00 p.m., reminder emails were sent to participants who had yet to complete their daily survey. Each daily survey took approximately 5–7 min to complete and included measures of perceived stress, loneliness, and marital satisfaction. Participant responses were automatically recorded in SurveyMonkey and securely stored on password-protected servers. At the conclusion of the study, the data were downloaded by the research team for analysis.

In total, 155 couples completed the daily survey. On average, spouses were married for 19.76 years (SD = 12.26). The majority of couples (66.5%) had between one and three children, with 24.5% reporting no children and 7.8% reporting four or more children. Most participants reported that they were Caucasian (88%), employed full-time, and had an average annual household income between $75,000 and $100,000. Data on participants age were not collected given the strong correlation with relationship duration (i.e., people of higher age have often been in the same relationship for a much longer time than people of younger age). Additionally, relationship duration tends to be more strongly associated with relationship outcomes when considered simultaneously with age (Bühler et al., 2024).

### 2.2 Measures

Consistent with past research employing intensive longitudinal designs (e.g., Silvia and Cotter, 2021), we selected well-validated, brief measures to minimize participant burden and fatigue. On day 1 of the survey, spouses completed demographic measures, whereas on days 2 to 8, participants completed daily questionnaires on stress, loneliness, and marital satisfaction.

#### 2.2.1 Perceived stress

Perceived stress was measured using four items developed by Cohen et al. (1983) that assessed the extent to which participants perceived their lives as unpredictable, uncontrollable, and overloaded. Participants were asked to reflect on the previous 24 h and to rate each of the following items using a 5-point scale (0 = *never*; 4 = *very often*): (1) not being able to control important things in life; (2) level of confidence in being able to handle personal problems; (3) feeling like things were going their way; and (4) having difficulties pile up to the point of not being able to overcome them. These items have been validated based on large probability samples (Cohen and Williamson, 1988) and have been widely administered in studies employing daily diary designs (e.g., Helgeson et al., 2015; Kumar et al., 2022). Responses to these items were subsequently summed (the second and third items were reverse coded), with higher scores representing higher daily perceived stress. Reliability estimates for each day were adequate for both husbands (*α* = 0.73 to 0.75) and wives (*α* = 0.74 to 0.78).

#### 2.2.2 Daily loneliness

Loneliness was assessed with a 3-item version of the UCLA Loneliness scale created by Hughes et al. (2004). Using a three-point scale (1 = *hardly ever*; 2 = *some of the time*; 3 = *often*), participants responded to the following items: (1) “Today, I lacked companionship”; (2) “Today, I felt left out”; and (3) “Today, I felt isolated from others.” Drawing from large adult-population samples, Hughes et al. (2004) found that these items had acceptable internal consistency (Cronbach’s *α* = 0.72) while demonstrating strong convergent validity with the original Revised-UCLA Loneliness scale. Similar psychometric properties have been observed in previous daily diary studies (e.g., Brown et al., 2023). Ratings across all items were summed to reflect greater loneliness. Reliability estimates for each day were appropriate for both husbands (*α* = 0.86 to 0.92) and wives (*α* = 0.82 to 0.90).

#### 2.2.3 Daily marital satisfaction

Marital satisfaction was measured with the Kansas Marital Satisfaction Scale (Schumm et al., 1983). The scale consists of 3 items that ask participants to report on a Likert-type scale (1 = *extremely dissatisfied*; 7 = *extremely satisfied*) the extent to which they were satisfied with (1) their marriage; (2) their spouse; and (3) their relationship with their spouse. Prior research has provided evidence for reliability and validity for the measure across various types of longitudinal designs (Bühler and Orth, 2022; Walsh and Neff, 2020). All scales were summed within each day to provide a total score with higher scores indicating greater marital satisfaction. Internal consistency of daily reports was high for both husbands and wives (*α* = 0.97 to 0.99).

#### 2.2.4 Control variables

Several demographic variables were included as control variables based on prior research demonstrating their associations with marital satisfaction. During the baseline survey on Day 1, participants reported on relationship duration and total number of children they had with their spouse as open-ended numeric responses. They also reported on their household income using an ordinal scale (1 = *less than $25,000*; 2 = *$25,001 to $50,000*; 3 = *$50,001 to $75,000*; 4 = *$75,001 to $100,000*; 5 = *$100,001 and above*). These variables were controlled given established links between relationship duration, presence of children, household income, and marital satisfaction (Karney, 2021; Twenge et al., 2003). We also controlled for study day to account for potential day-of-week effects on stress and fatigue across the seven-day reporting period (Sliwinski et al., 2009). Study day was centered so that the first diary day (Day 2) was 0, the second diary day (Day 3) was 1, continuing through Day 8, which was 6.

### 2.3 Analysis

Preliminary analyses included evaluation of missingness, bivariate Pearson correlations between the main study variables, as well as paired mean difference tests between husbands’ and wives’ reports. Participants submitted 1,426 daily diary surveys across the study period, with an average of 4.6 surveys per participant (SD = 2.6). Among surveys that were submitted, 96.7% contained complete data on stress, loneliness, and marital satisfaction. Approximately 78.4% of participants completed six or more valid diary days. Importantly, there were no significant differences between participants who completed at least one valid diary day and those who completed none on household income, *t*(48.6) = 0.33, *p* = 0.739, years married, *t*(55.3) = −0.22, *p* = 0.829, or number of children, *t*(58.4) = −0.69, *p* = 0.495. Prior-day stress, loneliness, and marital satisfaction did not predict whether the next day’s survey was completed (all *p*s > 0.15). Paired-sample *t*-tests comparing husbands and wives revealed no significant differences in the number of valid diary days completed, *t*(154) = −0.41, *p* = 0.681, mean daily stress, *t*(108) = 0.54, *p* = 0.593, mean daily loneliness, *t*(109) = −1.43, *p* = 0.156, or mean daily marital satisfaction, *t*(108) = −0.30, *p* = 0.764.

For household income, length of marriage, and number of children, a couple-level score was created by averaging each spouse’s responses. As is standard practice, multilevel modeling was used to account for the nested nature of the data (i.e., days nested within individuals and individuals nested within couples), and to examine between-person and within-person associations among daily stress, daily loneliness, and daily marital satisfaction (Bolger and Laurenceau, 2013). Consistent with recommendations for multilevel modeling (Hamaker, 2025), we first calculated intraclass correlation coefficients (ICC) to assess whether variance in marital satisfaction existed at both the between-person and within-person levels. *R^2^* values were calculated following the recommendation of Nakagawa and Schielzeth (2013), which contrasts marginal R*^2^* with conditional *R^2^* values to indicate the amount of variance explained by across modeled effects.

We estimated five sequential models using a nested model-building strategy. After establishing baseline variance structure (Model 1) and controlling for demographics (Model 2), we tested whether stress and loneliness independently predict marital satisfaction (Models 3 and 4) before examining whether loneliness moderates the association between daily stress and marital satisfaction (Model 5). Our approach tests whether the substantive findings hold when accounting for demographic variables and main effects. Both within and between-person predictors of stress and loneliness were included in the models. Specifically, a person-mean-centered, within-person predictor was included (i.e., one’s deviation on a given day from their personal average across the 7 days), as well as a grand-mean-centered, between-person predictor (i.e., a person’s average across the 7 days). Multilevel modeling analyses were estimated in SAS (Proc Mixed, Rstudio nmle package), and missing data was handled using restricted maximum likelihood.

## 3 Results

Descriptive statistics and bivariate Pearson correlations can be found in Table 1. Although most participants reported modest levels of stress and loneliness as well as high levels of marital satisfaction, no variable demonstrated a significant departure from normality that could potentially alter study findings. Paired sample *t*-tests were also conducted to identify potential spousal differences across study variable means. Results showed that husbands in the sample reported higher levels of loneliness than wives (*t* (434) = −2.49, *p* < 0.01); however, husbands and wives did not differ in their reports of stress or daily marital satisfaction. As shown in Table 1, between-person loneliness, stress, and marital satisfaction were correlated in expected directions. For both husbands and wives, higher loneliness was associated with higher stress and lower marital satisfaction. Higher stress was also associated with lower marital satisfaction for both spouses. These correlations were generally moderate in size, except for the association between loneliness and marital satisfaction, which was large.

**Table 1:** Descriptive statistics and bivariate correlations for study variables.

	Loneliness	Stress	Marital satisfaction	Income	Years married	Number of children
1. Loneliness	**0.34**	**0.49**	**−0.64**	**−0.25**	0.03	−0.06
2. Stress	**0.49**	**0.24**	**−0.39**	**−0.25**	0.02	−0.01
3. Marital satisfaction	**−0.63**	**−0.45**	**0.30**	**0.24**	−0.03	−0.01
4. Income	−0.08	0.00	**−0.10**	a	**0.17**	**0.15**
5. Years married	0.06	−0.06	−0.06	**0.17**	a	0.62
6. Number of children	−0.02	−0.07	0.05	**0.15**	0.62	a
Wife mean (SD)	3.75 (1.34)	3.62 (3.44)	17.38 (4.88)	4.40 (1.17)	19.77 (12.26)	1.85 (1.36)
Husband mean (SD)	3.94 (1.47)	3.57 (3.11)	17.27 (4.99)	a	a	a
Range	3 to 9	0 to 15	3 to 21	1 to 6	0 to 65	0 to 6

All correlations reflect between-person associations calculated using each spouse’s summed score across the daily reports. Pearson correlations above the diagonal represent bivariate associations among wives’ variables; correlations below the diagonal represent bivariate associations among husbands’ variables. Correlations on the diagonal represent associations between husband and wife reports of the same variable (e.g., husband loneliness with wife loneliness). a = variables measured at the couple level, so these are equal for husbands and wives. Bold values are statistically significant at *p* < 0.05.

As standard practice, we first examined the variability across various levels of the data by calculating intraclass correlation coefficients from models with no predictors (ICC; Hamaker, 2025). Results indicated that 66% of the variance in marital satisfaction in the current sample was due to between-person differences, for both husbands and wives, and some variability (33%) in marital satisfaction across days. This within-person variability suggests that spouses’ evaluations of their marriage varied across the diary period, supporting the use of daily assessments to examine short-term changes in marital satisfaction. The observed ICCs are consistent with those found in daily diary studies and confirm the use of multilevel modeling (Bolger and Laurenceau, 2013).

In regard to the multilevel results, each predictor was decomposed into between-person predictors represent how a person’s average differs from other respondents’ averages (i.e., intra-individual differences) and within-person predictors represent daily fluctuations from one’s average (i.e., inter-individual changes). As seen in Model 3 of Table 2, the results were consistent with our main effects hypotheses. Both between-person and within-person predictors of daily stress for husbands and wives were linked to their respective daily marital satisfaction scores, with income, years married, number of children, and time (days) controlled in the model. In Model 4, main effects of loneliness are added, and results show that both between- and within-person reports of loneliness for wives were linked to lower marital satisfaction. Among husbands, only between-person effects of loneliness were linked to marital satisfaction.

**Table 2:** Unstandardized actor associations between daily stress and marital satisfaction, as moderated by loneliness (*N* = 155 couples).

	**Model 1**	**Model 2**	**Model 3**	**Model 4**	**Model 5**
**Wives**					
Intercept	17.34***	17.25***	17.42***	17.13***	16.68***
Income		1.20***	0.66*	0.24	0.23
# of children		-0.14	-0.11	-0.46	-0.43
Years married		-0.03	-0.02	0.03	0.03
WP-stress			-0.45***	-0.27***	-0.26***
BP-stress			-0.51***	-0.07	-0.04
WP-lonely				-1.22***	-1.23***
BP-lonely				-2.72***	-3.05***
WP-stress*WP-lonely					0.04
BP-stress*BP-lonely					0.24*
**Husbands**					
Intercept	17.31***	16.99***	17.14***	17.42***	17.30***
Income		0.60	0.22	-0.32	-0.27
# of children		0.24	0.29	0.20	0.19
Years married		-0.05	-0.07	-0.03	-0.02
WP-stress			-0.33***	-0.25***	-0.25***
BP-stress			-0.86***	-0.28**	-0.28**
WP-lonely				-0.25	-0.24
BP-lonely				-2.54***	-2.62***
WP-stress*WP-lonely					0.13*
BP-stress*BP-lonely					0.02
**Model information**					
# of parms/N of days	6/1,418	13/1,281	17/1,259	21/1,247	25/1,247
-2LL	7,619.10	6,871.23	6,649.82	6,375.97	6,378.01
AIC	7,631.10	6,897.23	6,683.82	6,417.97	6,428.01
Wife BP variance	17.01	14.60	12.12	6.47	6.09
Husband BP variance	16.54	17.54	11.62	5.12	5.13
Marginal *R*^2^/conditional *R^2^*	-	-	-	0.39/0.77	0.39/0.77

WP = within-person. BP = between-person. REML = restricted maximum likelihood. Study day was coded 0 through 6. * *p* < .05. ** *p* < .01. *** *p* < .001. Study day was coded as a linear time variable (0 = first diary day through 6 = last diary day) and was controlled in Models 2 through 5. For wives, study day was significant in Model 2 (*b* = 0.179, *p* = 0.006) and became non-significant after within-person stress was added in Model 3 (*b* = 0.085, *p* = 0.182), remaining non-significant in Models 4 and 5. For husbands, study day was non-significant across all models (*p*s > 0.48). *p < 0.05. **p < 0.01. ***p < 0.001.

**Figure 1:**
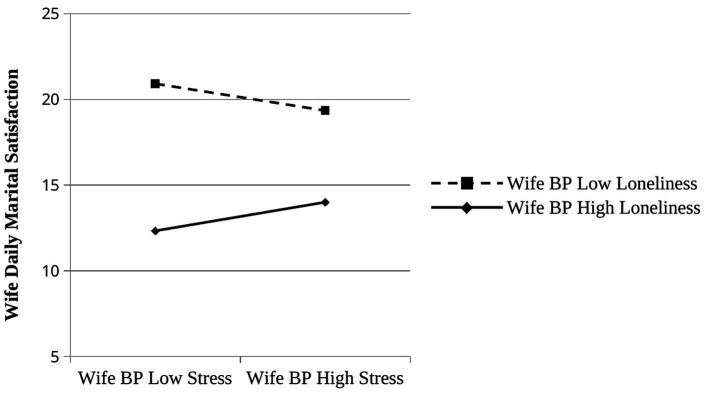
Interaction Effects between Wives’ Daily Loneliness and Daily Stress on their Reports of Daily Marital Satisfaction. BP, between person; high and low values represent ± 1 standard deviation from the BP mean.

As seen in Model 5, wives’ between-person stress and loneliness were associated with daily marital satisfaction. Among wives who reported low loneliness (1 *SD* below the mean), higher between-person stress was significantly associated with lower daily marital satisfaction (*b* = −0.272 [−0.53, −0.01], *t* = −2.06, *p* = 0.040). By contrast, among wives who reported high loneliness (1 *SD* above the mean), the association between between-person stress and daily marital satisfaction did not reach statistical significance (*b* = 0.187 [−0.09, 0.47], *t* = 1.31, *p* = 0.190) (see Figure 1). The gap between the two lines illustrates the main effects of loneliness on marital satisfaction.

**Figure 2:**
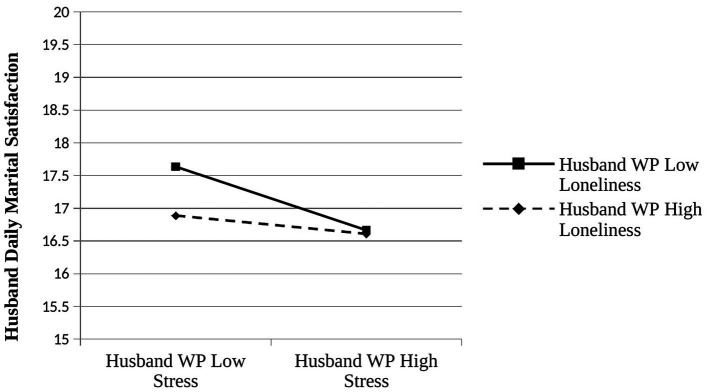
Interaction Effects between Husbands’ Daily Loneliness and Daily Stress on their Reports of Daily Marital Satisfaction. WP, within-person; high and low values represent ± 1 standard deviation from the WP mean.

Also seen in Model 5, the interaction between husbands’ within-person stress and loneliness was significantly associated with their own daily marital satisfaction. As seen in Figure 2, on days when husbands reported lower than usual loneliness (1 SD below the person mean), higher daily stress was significantly associated with lower daily marital satisfaction (*b* = −0.338 [−0.50, −0.18], *t* = −4.13, *p* < 0.001). Alternatively, on days when husbands reported higher than usual loneliness (1 SD above the person mean), daily stress was not significantly associated with marital satisfaction (*b* = −0.133 [−0.29, 0.03], *t* = −1.63, *p* = 0.102).

Analyses were also conducted to explore partner effects of loneliness as a moderator of the stress to marital satisfaction link, as well as exacerbated actor-partner interactive effects. We explored whether husband loneliness moderated the effect of the wife’s stress on her own marital satisfaction, and whether wife loneliness moderated the effect of the husband’s stress on his own marital satisfaction. No significant partner effects from these models were found (see Table 3).

**Table 3:** Unstandardized partner associations between daily stress and marital satisfaction, as moderated by loneliness (*N* = 155 couples).

	**Model 1**	**Model 2**	**Model 3**	**Model 4**	**Model 5**
**Wives**					
Intercept	17.34***	17.25***	17.24***	17.13***	16.68***
Income		1.20***	1.09*	0.24	0.23
# of children		-0.14	-0.14	-0.46	-0.43
Years married		-0.03	-0.02	0.03	0.03
WP-stress			-0.45***	-0.27***	-0.26***
BP-stress			-0.51***	-0.07	-0.04
Partner WP-stress				-0.13	-0.13
Partner BP-stress				0.04	0.04
WP-lonely				-1.22***	-1.23***
BP-lonely				-2.72***	-3.05***
Partner WP-lonely				0.03	
Partner BP-lonely				-0.25	
WP-stress*WP-lone					0.04
BP-stress*BP-lone					0.24
Partner WP-stress*WP-lone					-0.10
Partner BP-stress*BP-lone					-0.08
**Husbands**					
Intercept	17.31***	16.99***	16.97***	17.30***	17.16***
Income		0.60	0.12	-0.31	-0.26
# of children		0.24	0.29	0.08	0.10
Years married		-0.05	-0.05	-0.01	-0.01
WP-stress			-0.27**	-0.27**	-0.28**
BP-stress			-0.72***	-0.20	-0.20
Partner WP-stress			-0.03	-0.05	-0.06
Partner BP-stress			-0.26	-0.07	-0.09
WP-lonely				-0.04	-0.12
BP-lonely				-2.65***	-2.67***
Partner WP-lonely				0.11	0.05
Partner BP-lonely				0.19	0.17
WP-stress*WP-lone					0.11
BP-stress*BP-lone					0.03
Partner WP-stress*WP-lone					0.08
Partner BP-stress*BP-lone					-0.03
**Model information**					
# of parms/N of days	6/1,418	13/1,281	21/771	29/752	37/752
-2LL	7,619.10	6,871.23	4,070.11	3,809.98	3,825.40
AIC	7,631.10	6,897.23	4,112.11	3,867.98	3,899.40
Wife BP variance	17.01	14.60	11.24	5.13	5.19
Husband BP variance	16.54	17.54	10.27	4.45	4.59
Marginal *R*^2^/conditional *R^2^*	-	-	-	0.40/0.75	0.40/0.76

WP = within-person. BP = between-person.* *p* < .05. ** *p* < .01. *** *p* < .001.

## 4 Discussion

Leveraging daily diary data collected from 155 couples, the present study examined associations among stress, loneliness, and marital satisfaction. The results were generally consistent with prominent Vulnerability-Stress-Adaptation Model (Karney and Bradbury, 1995; McNulty et al., 2021), with results from the main effects models showing that higher levels of daily stress (Model 3) and loneliness (Model 4) each coincided with lower marital satisfaction for both husbands and wives. Gender differences were observed for the tested interaction between stress and loneliness (Model 5). For wives, the interaction at the between-person level revealed that when higher levels of stress coincided with lower levels of loneliness, they tended to report lower levels of marital satisfaction. For husbands, the interaction at the within-person level showed that the negative association between stress and marital satisfaction was more pronounced on days when husbands reported lower loneliness (relative to their own average) than on days when loneliness was higher.

### 4.1 Variance decomposition and correlations

Because the variance decomposition and bivariate correlations provide context for interpreting the daily diary findings, we will first discuss these findings. The variance decomposition from the unconditional models indicated that approximately two-thirds of the variance in marital satisfaction was attributable to stable differences between persons, whereas the remaining one-third reflecting day-to-day fluctuation within person, which is consistent with past research showing that between-person effects may be more prominent than within-person associations (Scheling et al., 2025; Totenhagen et al., 2016). It is also important to note that the bivariate correlations between spouses were modest in size. Recent evidence on interdependence within couples suggests that similarity between spouses may be more modest and more variable across constructs than is often assumed (Horwitz et al., 2023; Jackson et al., 2014; Sels et al., 2020), and these correlations support that conclusion. For loneliness in particular, the modest spousal correlation aligns with cognitive frameworks positing that loneliness arises from individually perceived discrepancies between desired and actual social connection (Perlman and Peplau, 1981) rather than from shared relational conditions that both partners would appraise similarly.

### 4.2 Main effects for stress and loneliness

The variance decomposition from the unconditional models indicated that approximately two-thirds of the variance in marital satisfaction was attributable to stable differences between persons, with the remaining one-third reflecting day-to-day fluctuation within persons. Moving onto our hypothesized associations, our findings revealed robust main effects for both stress and loneliness in their association with marital satisfaction. In regard to stress, negative associations emerged between stress and marital satisfaction at the between-person and within-person levels (Model 3). At the between-person level, spouses who reported higher average levels of daily stress across the study period reported lower marital satisfaction compared to those experiencing less stress. The within-person analyses showed that on days when spouses experienced greater stress than usual, they tended to report lower marital satisfaction. Our findings parallel recent research demonstrating negative associations between daily stress and spouses’ evaluations of their marriage (Neff and Buck, 2023; Randall and Bodenmann, 2017). It may be that stress levels lowered self-regulation capacity among respondents (Buck and Neff, 2012), more negative mood (Falconier et al., 2014) or attributions partners made about each other’s words and actions (Neff and Karney, 2004).

Drawing on cognitive theories of loneliness, we also expected main effects for loneliness such that spousal reports of daily loneliness would be associated with lower marital satisfaction at the between-person and within-person levels. At the between-person level, both husbands and wives who reported higher average levels of loneliness across the study period reported lower marital satisfaction compared to those experiencing less loneliness (Model 4). Our finding generally aligns with prior cross-sectional and longitudinal research showing that higher levels of loneliness, on average, were associated with lower levels of relationship satisfaction (Frye-Cox and Hesse, 2013; Mund and Johnson, 2021).

At the within-person level, only wives’ daily fluctuations in loneliness were associated with their own marital satisfaction, indicating that on days when wives experienced greater loneliness than their average levels, they reported lower marital satisfaction (Model 4). The lack of statistically significant findings for husbands at the within-person level may reflect gender differences in how loneliness manifests throughout daily interactions. Prior research has shown that women, compared to men, may be more relationship-focused and attuned to changes in their relationships (e.g., Kiecolt-Glaser and Newton, 2001). As a result, sensitivity to relationship changes may correspond to wives more readily incorporating experiences of social disconnection into their evaluations of marriage. The observed within-person association between loneliness and marital satisfaction among wives extends emerging evidence about the broader significance of daily variations in loneliness for individual well-being (Witzel et al., 2024). Although our findings identify daily stress and daily loneliness as correlates of daily marital evaluations, when examined simultaneously our results provide a more nuanced view as to how stress and loneliness are associated with marital satisfaction.

### 4.3 Interactive effects of stress and loneliness

We also hypothesized that the experience of daily loneliness would strengthen the negative association between stress and marital satisfaction for husbands and wives; however, we found mixed support for such an interaction effect (Model 5). Contrary to our expectations, at the between-person level we found that when wives reported both higher average levels of stress and loneliness, they tended to report *higher* levels of marital satisfaction. Although unexpected, these results are grounded in prior research on social withdrawal and sex differences in reliance on social networks.

In regard to the between-person interaction effect of stress and loneliness for wives, it could be the case that when wives perceive fewer external sources of support, they may rely on their husbands to help alleviate their stress, thereby coinciding with more favorable reports of daily marital satisfaction. That is, wives who reported higher average stress and higher average loneliness may have differed from other wives in the extent to which they relied on their spouse for support. Prior work has shown that wives may rely on their spouse more when under stress (Neff and Karney, 2005), and they may benefit most from spousal support when support from friends and family is limited (Finkel et al., 2014; Wilcox and Dew, 2012). Alternatively, it is also plausible that wives with higher average stress and loneliness were more likely to seek support when experiencing stress, which may be related to higher marital satisfaction. This explanation is consistent with tend-and-befriend theory, which suggests that women may be especially likely to seek affiliation under conditions of stress (Taylor et al., 2000). We can only speculate, however, about whether wives received support from their husbands, family members, friends, or other members of their social networks. It is plausible that wives’ marital satisfaction may have been preserved by support received within the marriage, support received outside the marriage, or support received across multiple relationships. Future researchers will have to explore this further by examining whether the source of support explains the association between stress, loneliness, and marital satisfaction.

This interaction should also be interpreted in light of possible statistical artifacts that warrant consideration given the unexpected direction of the effect. For instance, the truncated response range for loneliness could have contributed to floor effects given that 65.9% of the daily responses. Because floor effects in the predictor restrict variance, floor effects would likely attenuate the interactive effect estimate and thereby making it more difficult to detect. Nevertheless, our explanation for this finding is speculative and should be interpreted with caution until replicated in future research.

With regard to husbands, their own reports of fluctuations in daily stress and loneliness interacted to predict marital satisfaction. Loneliness appeared to play an important role on days with low stress such that husbands with low loneliness reported much higher marital satisfaction. On days with high stress, husbands with either low or high loneliness did not differ in their reports of marital satisfaction. One interpretation of this finding is that husbands’ need for affiliation and support may coincide with high stress days, which is consistent with tend-and-befriend theory (Taylor et al., 2000). In a review of the literature on sex differences in response to stress, Taylor et al. (2000) concluded that under conditions of stress, the desire to affiliate with others is substantially more marked among females than among males. Husbands have reported greater stress resulting from work and finances (Almeida and Kessler, 1998) and are more likely than wives to report more marital strife on days they experienced tensions at work (Bolger et al., 1989).

### 4.4 Limitations, strengths, and directions for future study

Findings from the current study are limited in a number of ways. First, couples were recruited through a convenience sampling approach, which resulted in a relatively homogenous non-representative sample of participants. More specifically, the sample gathered in the current study was predominately White and relatively affluent, and couples had been married, on average, for approximately 20 years, thereby limiting the generalizability of the results. Moreover, generally spouses reported relatively stable high levels of marital satisfaction, which may be an artifact of collecting dyadic data (Barton et al., 2020; Park et al., 2021), which may also influence generalizability of the findings. Considering the relatively low levels of stress and loneliness that were reported coupled with high marital satisfaction scores, it is plausible that our results underestimate the variable associations. Future research might involve purposive sampling of couples targeted for risk of high loneliness, or randomly selected couples from larger populations.

Second, because the present analyses were based on same day reports, the findings should be interpreted as associations and cannot establish temporal ordering among stress, loneliness, and marital satisfaction. Although our study was firmly grounded in stress in the Vulnerability-Stress-Adaptation model (VSA; Karney and Bradbury, 1995; McNulty et al., 2021) that position loneliness as an enduring vulnerability that may moderate the stress-marital satisfaction association, future research may seek to address the directionality of these associations. Nevertheless, the daily diary approach adds to the literature in an important way by considering both stable and varying aspects of loneliness in relation to the links between stress and marital satisfaction.

Third, adaptive processes, which are a key component of the VSA model, were not measured. According to the VSA model, vulnerabilities (e.g., loneliness) and stress interact to impact marital satisfaction, in part, by undermining adaptive processes such as couples’ capacity to engage in supportive communication or constructive problem-solving. Without data on adaptive processes, our understanding of how the interactive association between stress and loneliness may contribute to marital satisfaction remains incomplete. Future research that incorporates daily measures of adaptive processes would offer a more complete test of the VSA model.

Finally, the measures for stress and loneliness used in this study were designed to capture participants’ overall daily experiences of each construct, without asking participants to identify sources of those experiences. Therefore, the data for this study cannot distinguish whether stress and loneliness could be attributed to a partner, other people or events outside of the marriage, or a combination of both. Future research should extend our findings by examining whether stress and loneliness that emerge within marriage are more strongly associated with marital satisfaction than stress and loneliness that emerge from other social relationships or external events, as such work would clarify whether these sources carry distinct implications for how stress and loneliness may relate to marital satisfaction.

Findings from the current study may also have clinical implications. Clinicians may assist couples to reframe loneliness as a means to gain a stronger sense of belonging, which is consistent with past theorizing on loneliness as well as he finding that greater stress and loneliness were associated with greater marital satisfaction among wives (Conoley and Garber, 1985). The interactive effect observed for wives may highlight the need to consider addressing loneliness in relation to the broader marital context, as wives with higher average stress and loneliness may be especially likely to seek belonging and reassurance within the marriage. Clinicians may therefore help couples identify whether the marital relationship is functioning as a reliable source of support when wives report feelings of stress and loneliness. Given evidence that loneliness often fluctuates over time (e.g., Buecker et al., 2024; Witzel et al., 2024), clinicians may also help couples distinguish transient loneliness from more persistent loneliness that requires more direct intervention. Importantly, given the relatively affluent and predominantly White composition of the sample, the aforementioned clinical implications should be exercised with caution to couples from more racially, ethnically, and socioeconomically diverse backgrounds. In doing so, clinicians may be better positioned to strengthen support processes within the marriage.

### 4.5 Conclusion

Using a daily diary design with data from 155 dyads, our study examined whether loneliness moderated the association between daily stress and marital satisfaction. Our results show that the interaction between stress and loneliness may operate differently for husbands and wives. Unexpectedly, for wives, average levels of loneliness moderated the between-person stress-satisfaction association, with highly lonely wives maintaining satisfaction during periods of high stress. For husbands, daily fluctuations in loneliness moderated the within-person stress-satisfaction association, with loneliness strengthening the negative association between stress and daily satisfaction. Our findings suggest that chronic loneliness may coincide with wives’ overall vulnerability to stress, whereas momentary feelings of loneliness may be more salient for husbands’ daily marital experiences.

## Data Availability

The raw data supporting the conclusions of this article will be made available by the authors, without undue reservation.
